# Cholestyramine alleviates bone and muscle loss in irritable bowel syndrome via regulating bile acid metabolism

**DOI:** 10.1111/cpr.13638

**Published:** 2024-03-25

**Authors:** Ming Chen, Wei Wei, Yi Li, Siliang Ge, Junmin Shen, Jiayu Guo, Yu Zhang, Xiang Huang, Xinyu Sun, Dongliang Cheng, Huayong Zheng, Feifan Chang, Junyu Chen, Jiang Liu, Qinxiang Zhang, Tianjunke Zhou, Kang Yu, Peifu Tang

**Affiliations:** ^1^ Senior Department of Orthopedics The Fourth Medical Center of Chinese PLA General Hospital Beijing China; ^2^ National Clinical Research Center for Orthopedics Sports Medicine & Rehabilitation Beijing China; ^3^ Department of Clinical Nutrition, Peking Union Medical College Hospital Chinese Academy of Medical Science and Peking Union Medical College Beijing China; ^4^ Department of Orthopedic Surgery Second Affiliated Hospital of Harbin Medical University Harbin China

## Abstract

Irritable bowel syndrome (IBS) is a widespread gastrointestinal disorder known for its multifaceted pathogenesis and varied extraintestinal manifestations, yet its implications for bone and muscle health are underexplored. Recent studies suggest a link between IBS and musculoskeletal disorders, but a comprehensive understanding remains elusive, especially concerning the role of bile acids (BAs) in this context. This study aimed to elucidate the potential contribution of IBS to bone and muscle deterioration via alterations in gut microbiota and BA profiles, hypothesizing that cholestyramine could counteract these adverse effects. We employed a mouse model to characterize IBS and analysed its impact on bone and muscle health. Our results revealed that IBS promotes bone and muscle loss, accompanied by microbial dysbiosis and elevated BAs. Administering cholestyramine significantly mitigated these effects, highlighting its therapeutic potential. This research not only confirms the critical role of BAs and gut microbiota in IBS‐associated bone and muscle loss but also demonstrates the efficacy of cholestyramine in ameliorating these conditions, thereby contributing significantly to the field's understanding and offering a promising avenue for treatment.

## Graphical abstract

This study reveals that irritable bowel syndrome (IBS) contributes to bone and muscle loss through microbial dysbiosis and increased bile acid (BA) levels. Notably, administering cholestyramine ameliorates these effects by decreasing BA contents and modulating gut microbiota, underscoring its therapeutic potential in managing IBS‐induced musculoskeletal deterioration.

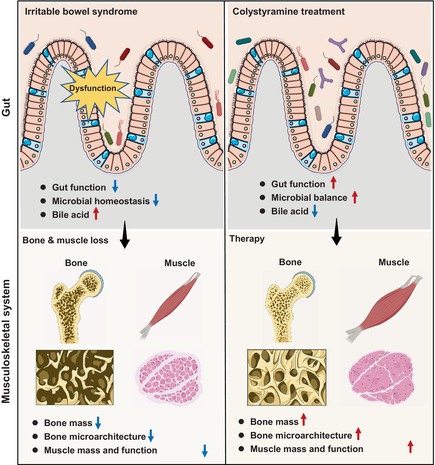

## 1 INTRODUCTION

Irritable bowel syndrome (IBS) is a prevalent functional gastrointestinal disorder characterized by recurrent abdominal pain and altered bowel habits. With IBS subtypes classified based on predominant faecal traits, IBS with predominant diarrhoea (IBS‐D) represents the most common, particularly in Western and Asian populations.^1^, ^2^ The pathogenesis of IBS is multifaceted, encompassing altered intestinal motility, visceral hypersensitivity, variations in gut wall compliance and psychological factors.^3^ Beyond these central manifestations, the scope and impact of its extraintestinal manifestations, especially on bone and muscle health, are not fully understood and warrant comprehensive exploration.

Recent advancements in research have begun to shed light on the potential extraintestinal impacts of IBS, particularly regarding musculoskeletal health. Emerging population‐based studies have started to draw a connection, suggesting a higher likelihood of osteoporosis among female IBS patients.^4^, ^5^ Additionally, a correlation between muscle loss or sarcopenia and IBS is gaining attention, often coinciding with osteoporosis to form a dual condition known as ‘osteosarcopenia’.^6^ While chronic inflammation is a recognized common denominator in gastrointestinal and musculoskeletal disorders,^7^, ^8^ the specific nuances of how IBS may influence bone and muscle integrity remain underexplored. This gap in understanding underscores the need for focused investigations into the skeletal implications of IBS.

Bile acids (BAs), catabolic end products of cholesterol, intricately involved in IBS pathophysiology. BAs not only have a direct antimicrobial effect but also significantly influence the gut microbiota composition and overall intestinal environment, affecting the mucosal barrier, immune activation and gut motility.^9^ In turn, gut microbiota regulates the biotransformation of BAs such as deconjugated of BAs and transformation from primary BAs to secondary BAs.^10^ What's more, BAs also play a pivotal role in gut functions which involve fluid transport, gut motility and visceral sensitivity.^11^ Notably, aberrations in BA profiles are observable in IBS patients,^12^ the therapeutic efficacy of BA sequestrants in managing IBS symptoms is well‐documented.^13^, ^14^ Intriguingly, there is emerging evidence linking BAs to musculoskeletal health. For example, studies in primary cholangitis patients have shown that lithocholic acid (LCA) adversely affects osteocyte viability and bone mineralization.^15^ Furthermore, exercise‐induced shifts in gut microbiota and BA metabolism have been linked to improved skeletal muscle glucose metabolism.^16^ These connections suggest a potential, albeit underexplored, role of BAs in the intersection of IBS and musculoskeletal disorders.

This study ventures into an in‐depth investigation of the potential interconnections among IBS, alterations in gut microbiota, changes in BA profiles and their collective impact on bone and muscle health. Through our detailed characterization of an IBS mouse model, we systematically explore how IBS contributes to bone and muscle loss, delving into the underlying mechanisms involving microbial dysbiosis and altered BA metabolism. A pivotal aspect of our research is the examination of increased BA levels in the faeces of mice with IBS and the subsequent impact on musculoskeletal health. We further assess the therapeutic potential of cholestyramine, a BA sequestrant, in this context. Our results demonstrate that the administration of cholestyramine not only ameliorates bone and muscle loss in IBS‐afflicted mice but also achieves this therapeutic effect by modulating the gut microbiota composition. These findings offer a novel understanding of the complex interplay between IBS, microbiota, BA metabolism and musculoskeletal health, highlighting new avenues for treatment strategies targeting IBS‐associated bone and muscle deterioration.

## 2 MATERIALS AND METHODS

### 2.1 Animals care and study approval

Male C57BL/6J mice, aged 8 weeks, were procured from Vital River Laboratories in Beijing, China. Before initiating experimental procedures, the mice underwent a 7‐day acclimation phase. They were housed in conditions of 25 ± 2°C, with 50 ± 5% humidity, under a 12‐h light/dark cycle. All procedures and treatments were conducted with the express approval of the Animal Care and Oversight Committee at the Chinese PLA General Hospital, Beijing, China (approval reference 2022‐X18‐11) and Peking Union Medical College Hospital, Beijing, China (approval reference XHDW‐2022‐125).

### 2.2 Chronic unpredictable mild stress (CUMS)‐induced IBS model

IBS mouse models were induced by exposure to the CUMS procedure.^17^, ^18^, ^19^ Briefly, CUMS‐treated mice were subjected to random chronic unpredictable stress for 49 consecutive days, including fasting for 12 h, water deprivation for 12 h, tilting, wet pressure for 12 h, flash illumination, tail‐pinch for 5 min, swimming in cold water for 5 min, bondage for 6 h, shake cages for 15 min. Nine different stress stimulations were randomly arranged in seven circles and each circle corresponded to a week of modelling. Detailed information is outlined in Table S1. For cholestyramine group, mice were intragastric administrated with cholestyramine (Medchemexpress, HY‐104081, 800 mg/kg/d) during the model induction period. On the last day, the animals were euthanized for the evaluation of intestinal motility, and samples from the colon, serum, bone and muscle were collected for subsequent analyses.

### 2.3 Evaluation of disease activity index (DAI), faecal water content and colon length

Throughout the experimental phase, we monitored the body weight (BW) of each mouse. Disease activity index (DAI) was evaluated using criteria such as body weight loss (scored from 0 to 4), stool consistency (0–3) and hematochezia (0–4), as outlined in Table S2. Upon euthanasia, we excised the colons, rinsed them with cold PBS and measured their lengths.

### 2.4 Abdominal withdrawal reflex assessment

To evaluate visceral hypersensitivity, we employed the abdominal withdrawal reflex (AWR) test, adapting a previously established method with minor modifications.^20^ We utilized a disposable paediatric silicon balloon‐urethral catheter. Mice underwent brief isoflurane anaesthesia, during which we inserted the catheter rectally up to the anus, securing it at the tail base to prevent displacement. Following full recovery from anaesthesia, mice were transitioned to a transparent observation cage for at least 30 min of acclimatization before testing commenced. Phasic distensions, increasing in intensity, were applied for 30‐s intervals with 4‐min rests. We assessed AWR using a semi‐quantitative scale: 0 indicated no response; 1, transient head movement followed by stillness; 2, abdominal muscle contraction without abdominal lift; 3, abdominal lifting; and 4, pronounced body arching alongside pelvic lifting.^21^

### 2.5 Measurement of diamine oxidase concentration

After collection, blood samples were centrifuged at 3000 rpm for 15 min at 4°C to obtain serum, which was stored at −80°C until further analysis. The serum diamine oxidase (DAO) concentration was measured using a validated enzyme‐linked immunosorbent assay (ELISA) kit (Vendor, City, State) to evaluate gut barrier function. The ELISA assay involved the use of specific anti‐DAO antibodies for capturing and detecting DAO in the serum samples. Standard curves were generated using known concentrations of DAO, and absorbance was measured at a wavelength of 450 nm with a microplate reader. All samples were assayed in duplicate, and the mean concentration was calculated.

### 2.6 Microcomputed tomography analysis

To assess bone density and structural intricacies, we employed the Inveon MM system (Siemens, Germany) for microcomputed tomography (micro‐CT). Specimens were scanned using the following parameters: pixel resolution of 8.89 μm, 60 kV voltage, 220 μA current and 1500 ms exposure across 360 rotations. This produced 1536 slices, each with voxels measuring 8.89 μm on every side. From the two‐dimensional images obtained, we generated three‐dimensional (3D) representations. For subsequent analysis, the Inveon Research Workplace (Siemens) was employed to calculate key bone metrics, including bone mineral density (BMD), bone volume to total volume ratio (BV/TV), trabecular number (Tb.N), bone surface to volume ratio (BS/BV), trabecular spacing (Tb.Sp) and trabecular thickness (Tb.Th) in the selected femoral region.

### 2.7 Dynamic histomorphometric analyses

Ten days before euthanasia, mice received an intraperitoneal injection of calcein (Sigma, C0875). This was followed by an injection of alizarin‐3‐methyliminodiacetic acid (Sigma, A3882) 3 days before euthanasia. Following euthanasia, femurs were extracted, subjected to 80% ethanol for fixation, and subsequently dehydrated. The bones were then prepared to yield undecalcified sections. We employed BioQuant software (OSTEO, version v20.8.60, BioQuant) to calculate both the mineral apposition rate (MAR) and the bone formation rate relative to the bone surface (BFR/BS).

### 2.8 Biomechanical assays

We assessed femoral biomechanical integrity using 3‐point bending tests, applying a uniform downward force of 1.0 mm/min with a servohydraulic testing system (Instron 4302, Norwood, MA, USA), consistent with standardized protocols. During testing, we continuously recorded load–deformation curves, concentrating on the femur's mid‐diaphysis. Our analysis quantified several indicators of biomechanical performance, including peak load, energy to peak load, Young's modulus, structural stiffness and fracture energy.

### 2.9 Immunofluorescence staining for bone

Femur samples were fixed in 4% paraformaldehyde at 4°C for 6 h, followed by decalcification in 15% EDTA. After thorough rinsing, samples were submerged in 30% sucrose, embedded in OCT and sectioned at 10 μm. Permeabilization was achieved with 0.5% Triton X‐100. Sections were then blocked in diluted goat serum (1:10) for 1 h and incubated with primary antibodies OSX (1:100, sc‐393325, Santa Cruz Biotechnology) and OPN (1:100, AF7665, Beyotime). Goat anti‐mouse IgG H&L Alexa 488 (1:100, ab150113, Abcam) was used as the secondary antibody. Nuclei were stained using a DAPI‐containing medium.

### 2.10 Muscle histology and immunofluorescence staining

Muscle specimens were encapsulated in OCT compound, flash‐frozen and sectioned at 10 μm. Haematoxylin and Eosin (H&E) staining involved a 25‐min haematoxylin incubation, a subsequent rinse and a 90‐s eosin submersion, followed by ethanol and xylene dehydration and Permount sealing for imaging. For immunofluorescence, fresh muscle samples were embedded in O.C.T., flash‐frozen in isopentane cooled with liquid nitrogen and sectioned to 10 μm thicknesses, then stored at −80°C. Sections were permeabilized and blocked with triton X‐100 and QuickBlock buffer (Beyotime, China) at room temperature for 30 min. They were incubated overnight at 4°C with primary antibodies: anti‐Dystrophin (ab15277, Abcam), anti‐MyHC I (BA‐D5, DSHB), anti‐MyHC IIa (SC‐71, DSHB) and anti‐MyHC IIb (BF‐F3, DSHB). Following several washes, sections were exposed to Alexa Fluor‐conjugated secondary antibodies (405, 488, 568 and 647) in QuickBlock buffer for 1 h at room temperature. Confocal microscopy captured the images, and the ImageJ software (NIH, USA) facilitated the quantification of both histological and immunofluorescence data.

### 2.11 qPCR

Total RNA was extracted using the RNA isolator Total RNA Extraction Reagent (Vazyme, R401). Reverse transcription was performed with 1 μg of total RNA, utilizing HiScript III RT SuperMix for qPCR (Vazyme, R323). Amplification reactions comprised a 20 μL volume containing 1 μL of cDNA, ChamQ Universal SYBR qPCR Master Mix (Vazyme, Q711) and specific primers, conducted in triplicate. The primer details are available in Table S3. Relative gene expression was quantified using the 2^−ΔΔCT^ method, normalized to the β‐actin gene.

### 2.12 BAs analysis

To mitigate sample degradation, faecal samples were initially thawed in an ice bath. Approximately 10 mg of each sample was then weighed and placed in a tube, along with 25 mg of zirconium oxide beads and 200 μL of an acetonitrile/methanol solution, which included 10 μL of internal standards. Following homogenization, the samples were centrifuged at 13,500*g* for 20 min at 4°C. A 10 μL aliquot of the supernatant was subsequently diluted with 90 μL of a mixture composed of equal parts acetonitrile/methanol and ultrapure water. After further centrifugation, the resulting supernatant was subjected to BA quantification using an ultraperformance liquid chromatography–tandem mass spectrometry (UPLC‐MS/MS) system (ACQUITY UPLC Xevo TQ‐S, Waters Corp., MA, USA). The mobile phases were 10 mmol/L ammonium acetate with 0.25% acetic acid (phase A) and a mixture of acetonitrile/methanol/isopropanol (phase B). The procedure employed a flow rate of 0.40 mL/min and a specified mobile phase gradient. The column temperature was held at 30°C, and the injection volume for each sample was 5 μL. Measurements were conducted with a capillary voltage of 2.0 kV in negative ion mode, a source temperature of 150°C, and a desolvation gas temperature of 550°C. UPLC‐MS/MS raw data files were processed via MassLynx software (v4.1; Waters, MA, USA) for peak integration, calibration and BA quantitation. All BA analyses were performed by Metabo‐profile Biotechnology (Shanghai, China).

### 2.13 Metagenomic analysis

In processing our raw Illumina sequencing data, we utilized Readfq (V8) to generate clean data for subsequent analysis. The MEGAHIT tool (v1.0.4‐beta) facilitated our metagenome assembly, yielding scaftigs, which were then segmented at N junctions. Open reading frames (ORFs) were predicted using MetaGeneMark for scaftigs longer than 500 bp, discarding sequences under 100 nt. The CD‐HIT software was instrumental in creating a preliminary non‐redundant gene catalogue. We then aligned each sample's clean data with this catalogue using Bowtie2, facilitating the determination of gene read counts. For species annotation, we matched Unigene sequences with entries in the NCBI's NR database through the DIAMOND tool. Dimensionality reduction was achieved using Krona charts, abundance summaries, clustering heatmaps, PCA and NMDS. We performed permutation tests between groups at each taxonomic level using Metastats, yielding p‐values that were subsequently adjusted to q‐values via the Benjamini and Hochberg False Discovery Rate method. The linear discriminant analysis (LDA) effect size (LEfSe) tool was employed for discriminative feature detection, using a default LDA score threshold of 3.5. Species were selected based on gradient presence, and a RandomForest model was constructed accordingly. For functional annotations, Unigene sequences were compared against the eggNOG and KEGG databases using the DIAMOND tool, and optimal Blast results were selected for further analysis.

### 2.14 Statistical analysis

Data analysis was conducted using the software GraphPad Prism, version 8.4.3 (GraphPad Software, California, USA). Values are expressed as mean ± standard deviation (SD). For comparisons, we employed either the two‐tailed Welch's *t*‐test or two‐way ANOVA. A *p*‐value less than 0.05 indicated statistical significance.

## 3 RESULTS

### 3.1 Characterization of IBS mouse model

**FIGURE 1:**
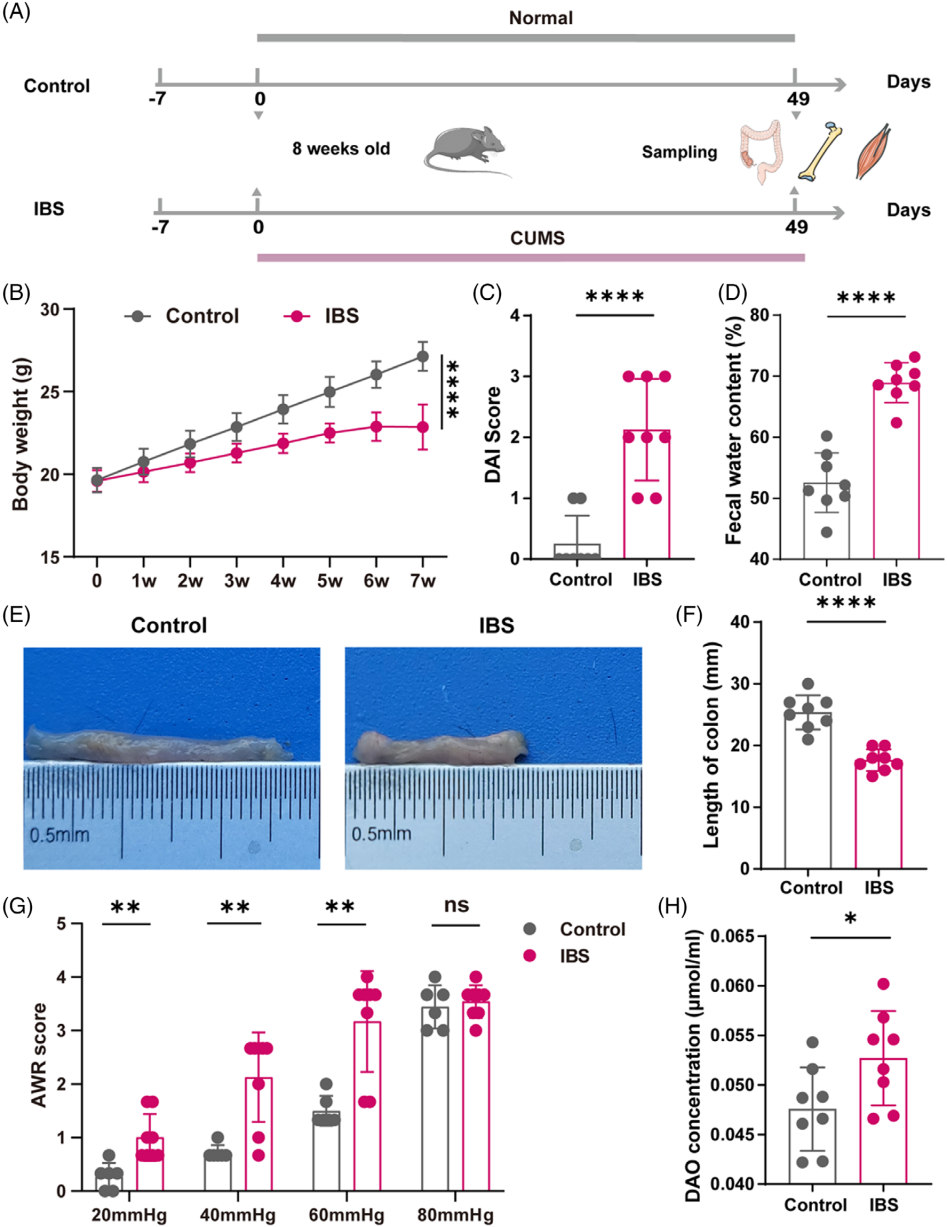
Characterization of phenotypes in IBS mice. (A) Schematic representation illustrating the experimental design. CUMS refers to the Chronic Unpredictable Mild Stress model. (B) Body weight change during the 7‐week experimental phase (*N* = 8). (C) DAI score evaluation (*N* = 8). (D) Faecal water content evaluation in mice (*N* = 8). (E,F) Representative images (E) and quantitative analyses of colon (F) (*N* = 8). (G) AWR score evaluation in mice (*N* = 8). (H) DAO content detection in mice (*N* = 8). Values are represented as the average ± standard deviation. The significance level (p value) was determined through a two‐sided Welch's *t*‐test. *p < 0.05; **p < 0.01; ****p < 0.0001.

For the induction of IBS in mice, we set up a chronic unpredictable mild stress (CUMS) model (Figure 1A). The results showed that those mice displayed a phenotype of the irritable bowel syndrome. Animals of the IBS model exhibited decreased body weight compared to the control, signalling a possible deterioration in general health (Figure 1B). Besides, the IBS model mice showed increased disease activity index (DAI) score (Figure 1C). Predominant in IBS‐D patients, diarrhoea was a key symptom replicated in our model, as evidenced by the increased water content in the faeces of the IBS group (Figure 1D). Anatomically, the IBS mice presented a conspicuous shortening of the colon (Figure 1E,F), suggestive of significant gastrointestinal alteration. Mice colon hypersensitivity was assessed by measuring the threshold intensity of the AWR score, which arises in response to colorectal distention (Figure 1G). At pressures of 20, 40 and 60 mmHg, the AWR was notably higher in the IBS model group compared to the control group, indicating augmented visceral hypersensitivity. However, an 80 mmHg pressure marked no discernable divergence between the groups, potentially due to the extremity of the stimulus provoking a universally intense response.^22^ Moreover, we analysed serum DAO levels, an enzyme produced by enterocytes in the intestinal villi, to gauge intestinal barrier integrity. Notably, its presence in the bloodstream surges upon compromised mucosal integrity. Our IBS mice registered a significant uptick in DAO levels compared to controls (Figure 1H), corroborating the presumption of impaired intestinal barriers. In summary, our data validate the successful replication of IBS in an experimental mouse model, offering a foundational platform for further pathological and therapeutic explorations.

### 3.2 IBS leads to bone and muscle loss in mice

**FIGURE 2:**
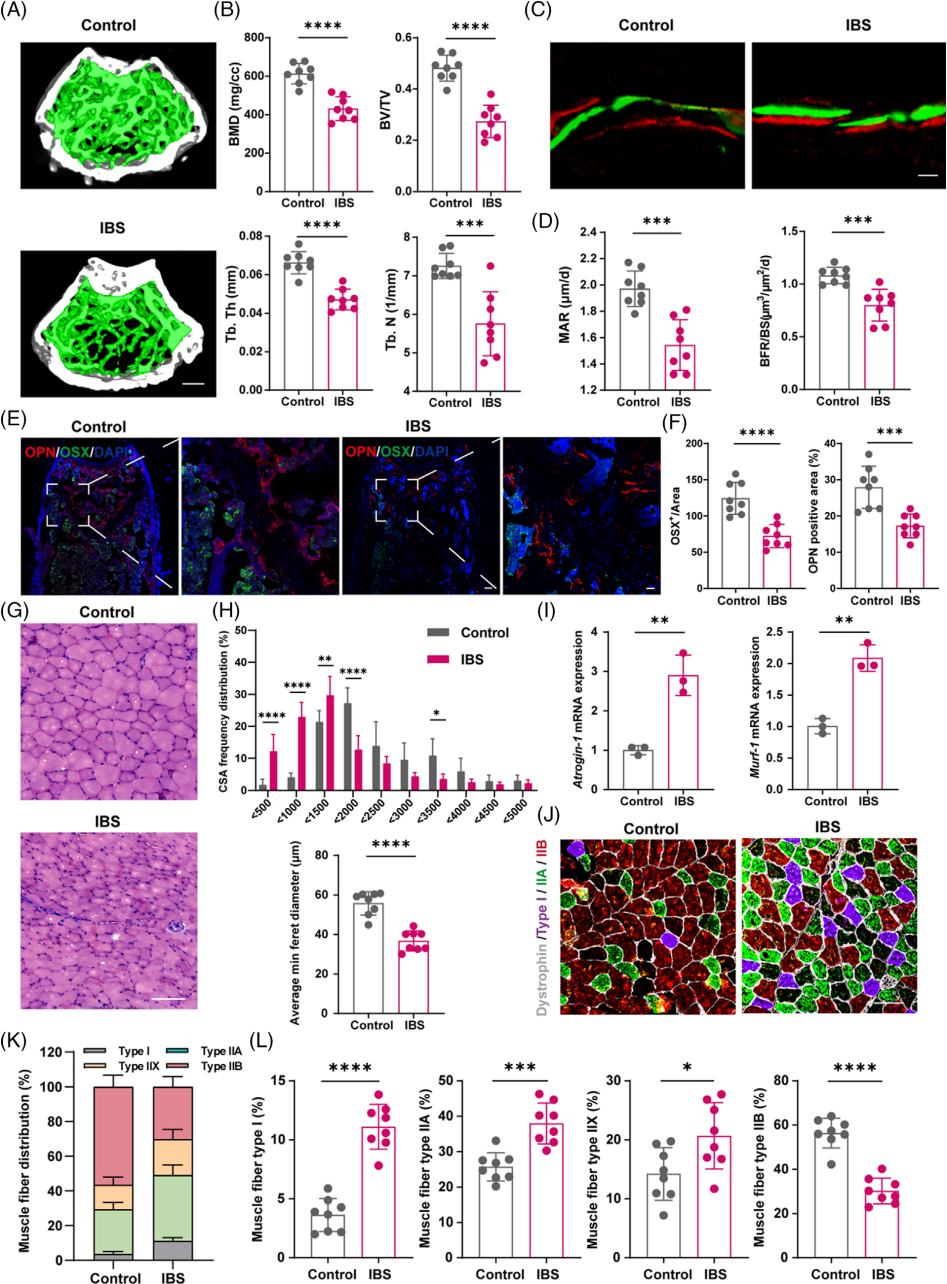
IBS leads to bone and muscle loss in mice. (A) Representative μCT images of distal femoral metaphyseal trabecular bone. (B) Quantitative analysis of bone mass, including BMD, BV/TV and Tb. Th and Tb. N (*N* = 8). Scale bar, 500 μm. (C,D) Representative images (C) and quantification (D) of new bone formation assessed by dynamic histomorphometric analyses (*N* = 8). Scale bar, 25 μm. (E,F) Representative images of OSX (green) and OPN (red) immunostainings (E) and quantification (F) of OPN^+^ and OSX^+^ area on distal femurs (*N* = 8). Scale bar, 100 μm and 25 μm, respectively. (G,H) Representative images of H&E staining in gastrocnemius cross‐sections (G), with quantification of average minimal Feret's diameter of myofibres and frequency distribution of CSA (H) (*N* = 8). Scale bar, 100 μm. (I) mRNA Expressions of *Atrogin‐1* and *Murf‐1* in gastrocnemius, tested by qPCR (*N* = 3). (J) Representative immunofluorescence images to visualize specific types of muscle fibres. Type I (purple), type IIA (green), type IIX (not shown) and type IIB (red). Scale bar, 100 μm. (K,L) Fibre type composition (K) and quantification (L) (*N* = 8). Values are represented as the average ± standard deviation. The significance level (*p* value) was determined through a two‐sided Welch's *t*‐test. *p < 0.05; **p < 0.01; ***p < 0.001; ****p < 0.0001.

To elucidate the repercussions of IBS on skeletal architecture, we undertook μCT examinations of mouse femurs, revealing a pronounced diminution in trabecular bone within the IBS subjects (Figure 2A). In addition, the IBS group displayed decreased bone mass including mineral density (BMD), bone volume/total volume (BV/TV), trabecular thickness (Tb. Th) and trabecular number (Tb. N) (Figure 2B), as well as increased bone surface area/bone volume (BS/BV) and trabecular spacing (Tb. Sp) (Figure S1). These results indicated that IBS condition deteriorates the bone structure. To assess the mechanical properties of bone, we conducted a mechanical stress test. We observed a significant reduction in maximum load, bone stiffness, Young's modulus and breaking energy in the IBS group (Figure S2). These findings collectively support that IBS negatively impacts bone structure, causing mechanical defects and bone fragility. Additionally, as indicated by bone histomorphometric analyses, the mineral apposition rate (MAR) and bone formation rate (BFR) of the IBS group were all significantly reduced, providing further evidence of impaired bone formation (Figure 2C,D). Through exploring the expression of two indicator proteins of osteoblasts, Osterix (OSX) and Osteopontin (OPN) in bone tissue, we conducted further research of bone formation activity. We performed immunofluorescence staining of femur samples and found that IBS reduced the numbers of OSX‐ and OPN‐positive osteoblasts in the femur of mice (Figure 2E,F). The data demonstrated a decrease in osteoblast function and bone mineralization in IBS‐induced mice.

Beyond skeletal impacts, we extended our investigation to IBS's influence on muscular health. Histological scrutiny, via H&E staining of gastrocnemius sections, unveiled pronounced irregularities in muscle fibre morphology within the IBS cohort, including dysmorphic myofibres and notable sarcolemma disruptions. This structural aberration was quantitatively substantiated by a diminished minimal Feret's diameter and a leftward shift in the cross‐sectional area (CSA) distribution curve, compared to controls (Figure 2G,H), evidencing the compromising effect of IBS on muscular architecture and fibre calibre. Corroborating the histological indicators of muscle atrophy, qPCR analyses identified a marked upregulation of atrophic markers, specifically *Atrogin‐1* and *Murf‐1* (E3 ubiquitin ligases), in the IBS subjects' gastrocnemius tissues (Figure 2I). Intrigued by the potential fibre type redistribution under IBS's pathophysiological stress, we employed fibre‐type‐specific immunofluorescence staining. Normal mice predominantly harboured type IIB fibres, succeeded by types IIA, IIX and I. However, IBS mice manifested a substantial reduction in type IIB fibres, counterbalanced by an upsurge in types I, IIA and IIX (Figure 2J–L). This shift from glycolytic type IIB fibres toward oxidative type I fibres underscores a metabolic reconfiguration, potentially contributing to the observed muscular deficits.

Synthesizing these findings, we assert that IBS precipitates not just skeletal fragility but also pervasive muscular atrophy, signifying a dual‐pronged degradation of musculoskeletal health. This comprehensive degradation necessitates an integrated understanding of IBS's systemic reach and a multidimensional approach to its management.

### 3.3 Microbial dysbiosis characterizes IBS‐induced bone and muscle loss

**FIGURE 3:**
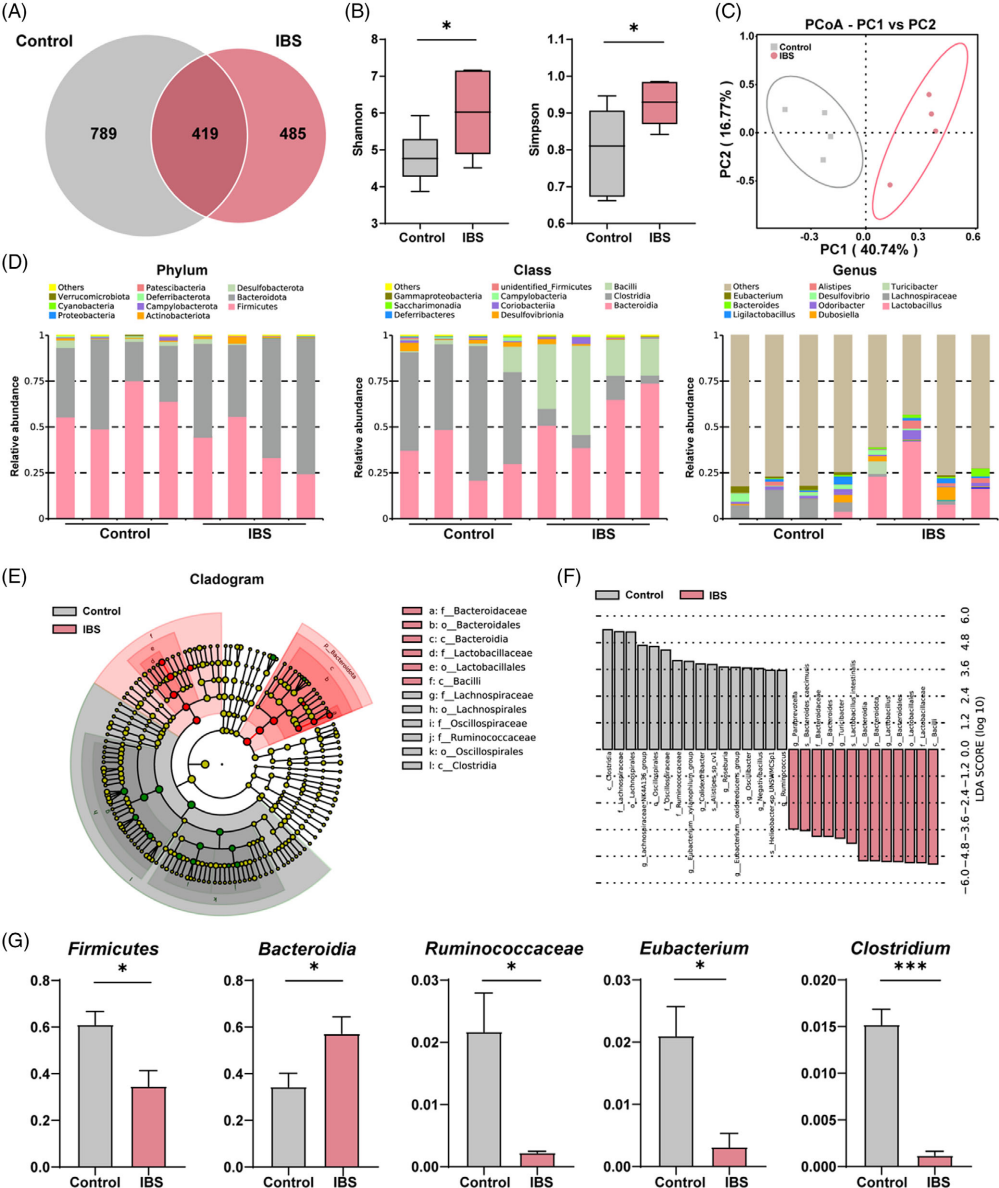
Metagenomic analysis reveals altered gut microbiota composition in IBS mice. (A) Venn diagram analysis of gene numbers detected in two groups. (B) The box plot illustrates *α*‐Diversity using the Shannon and Simpson indices. (C) Principal Coordinate Analysis (PCoA) of *β*‐diversity at the phylum tier is conducted via a Bray–Curtis matrix comparison for both groups. (D) Structure plot of the relative faecal bacterial abundances in phylum, class and genus‐level based on Bray–Curtis distance. (E,F) Analysis of Cladogram generated from LEfSe (E) and LDA score (F) across different taxa levels. (G) Quantitative analysis of differential taxa in two groups. Values are represented as the average ± standard deviation. The significance level (*p* value) was determined through a two‐sided Welch's *t*‐test. *p < 0.05; ***p < 0.001.

To elucidate the underlying microbiotic architecture in IBS‐associated bone and muscle degradation, we embarked on comprehensive metagenomic sequencing of faecal specimens from both control and IBS‐affected mice. Utilizing Illumina's high‐throughput sequencing, we instituted a Venn diagram assessment, revealing a pronounced genetic divergence between the IBS and control cohorts (Figure 3A). This disparity was further substantiated through *α*‐diversity (Shannon and Simpson indexes) (Figure 3B) and *β*‐diversity (principal‐coordinate analysis, PCoA) evaluations (Figure 3C).

Subsequent taxonomic scrutiny, spanning from phylum to species strata and pivoting on Bray–Curtis dissimilarity, unearthed significant microbial shifts. At the phylum level, IBS‐induced mice show a notable decline in *Firmicutes* and *Desulfobacterota*, and a marked rise in *Bacteroidota* and *Actinobacteriota* (Figure 3D, left). At the class level, a decreased abundance of *Clostridia*, *Campylobacteria* and *Desulfovibrionia* was observed, while *Bacilli*, *Bacteroidia* and *Coriobacteriia* showed increased abundance under IBS condition (Figure 3D, middle). At the genus level, we observed a diminished abundance of *Lachnospiraceae*, *Desulfovibrio* and *Eubacterium*, and an ascending abundance of genera including *Lactobacillus*, *Dubosiella*, *Alistipes* and *Bacteroides* in IBS group (Figure 3D, right).

Microbial community rearrangements were corroborated via LEfSe clustering (Figure 3E), with LDA scores accentuating a diminished representation of *Clostridia*, *Lachnospiraceae* and *Lachnospirales*, and an expanded footprint of *Bacilli*, *Lactobacillaceae* and *Bacteroidota* in IBS‐afflicted subjects (Figure 3F). Through quantitative analysis of differential taxa, we found significant increase in *Bacteroidia*, and notable decrease in *Firmicutes*, *Ruminococcaceae*, *Eubacterium* and *Clostridium* (Figure 3G). Notably, the taxa exhibiting pronounced contraction are recognized as pivotal constituents in BA transformation, signalling that BA dysregulation might indeed constitute a critical nexus in IBS pathology.

### 3.4 BAs are increased in the faeces of mouse CUMS model and human IBS cases

**FIGURE 4:**
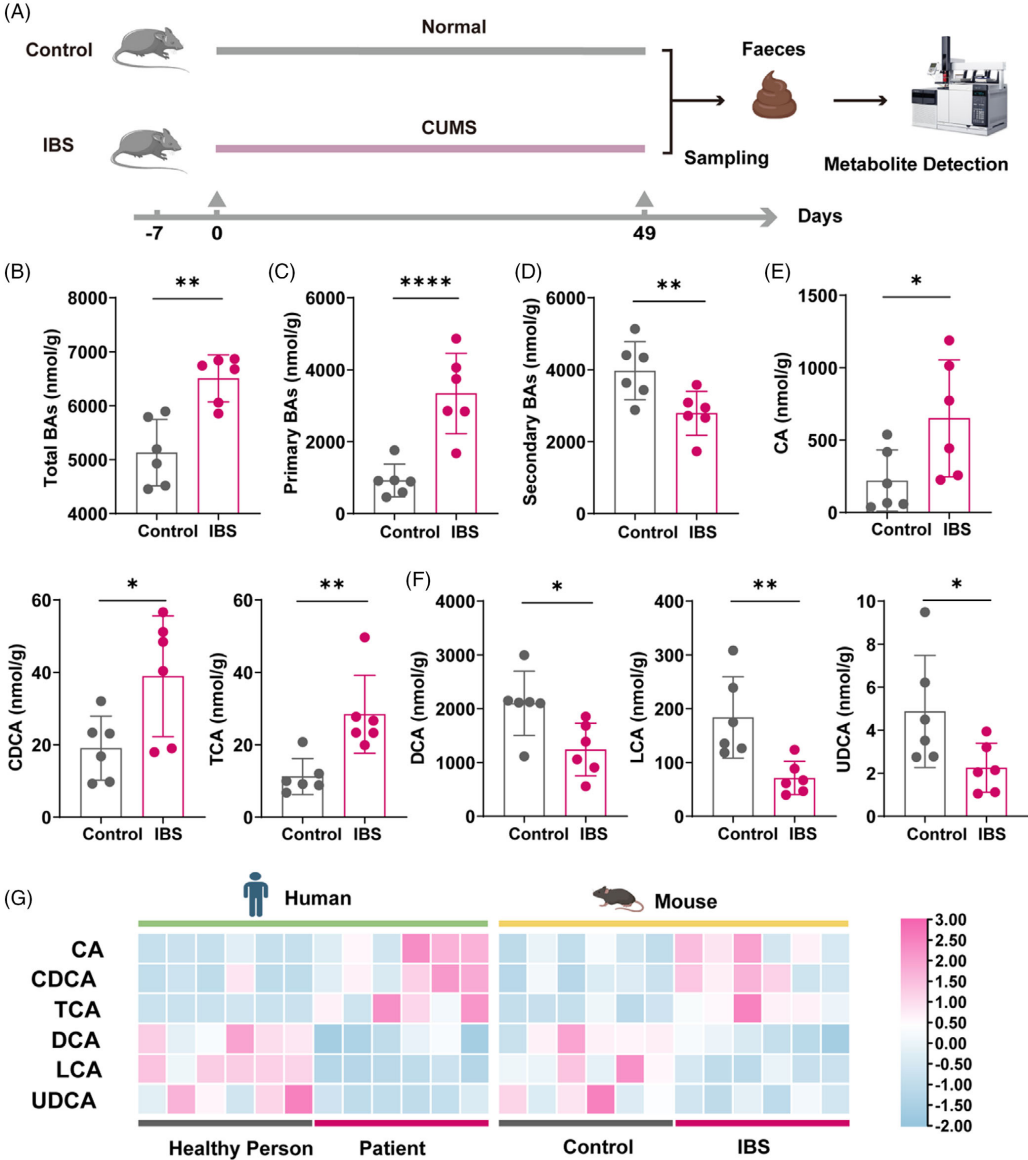
Bile acids (BAs) were increased in the faeces of mouse CUMS model and human IBS cases. (A) Schematic representation illustrating the experimental design. Bile acids were detected in faeces through UPLC‐MS/MS analysis. (B–D) Quantification of total BAs (B), primary BAs (C) and secondary BAs (D). (E) Quantification of cholic acid (CA), chenodeoxycholic acid (CDCA) and taurocholic acid (TCA). (F) Quantification of deoxycholic acid (DCA), lithocholic acid (LCA) and ursodeoxycholic acid (UDCA) (*N* = 6). (G) Side‐by‐side heatmap comparison of faecal BA elevation between mouse CUMS model and human IBS cases. Values are represented as the average ± standard deviation. The significance level (*p* value) was determined through a two‐sided Welch's *t*‐test. *p < 0.05; **p < 0.01; ****p < 0.0001.

Our preceding findings prompted us to hypothesize a critical role for BA metabolism in the pathogenesis observed in IBS‐afflicted mice. To test this, we employed UPLC‐MS/MS analysis to assess BA levels in faecal samples of both mouse models and control groups (Figure 4A). Notably, this analysis aligns with our team's prior research indicating elevated BA levels in human IBS cases.^23^ Confirming our expectations, IBS models exhibited a substantial surge in total faecal BAs relative to the control group (Figure 4B). Intriguingly, this increase was predominantly ascribed to primary BAs, with secondary BAs concurrently showing a reduction (Figure 4C,D). Further dissection of BA profiles revealed that the elevation was chiefly driven by primary BAs such as cholic acid (CA), chenodeoxycholic acid (CDCA) and taurocholic acid (TCA), while secondary BAs, including deoxycholic acid (DCA), LCA and ursodeoxycholic acid (UDCA), were in decline (Figure 4E,F). Furthermore, we expanded our investigation to include a comparative analysis of faecal BA profiles between the mouse CUMS model and human IBS‐D cases in our previous study.^23^ To this end, we generated a side‐by‐side heatmap that delineates BA levels across both species (Figure 4G). This analysis unveiled a consistent elevation in primary BAs such as cholic acid (CA), chenodeoxycholic acid (CDCA) and taurocholic acid (TCA) in both human IBS samples and the mouse model. Simultaneously, secondary BAs like deoxycholic acid (DCA), LCA and ursodeoxycholic acid (UDCA) manifested a decline. Notably, the parallel trends observed in both humans and mice not only corroborate our earlier human‐centric findings but also underscore a potentially conserved pathological mechanism underpinning IBS across species. Significantly, these BA alterations coincided with the detected shifts in gut microbiota, underscoring a potentially aberrant escalation in primary BAs amidst the microbial dysbiosis in IBS.

### 3.5 Reducing BA content relieves IBS phenotypes

**FIGURE 5:**
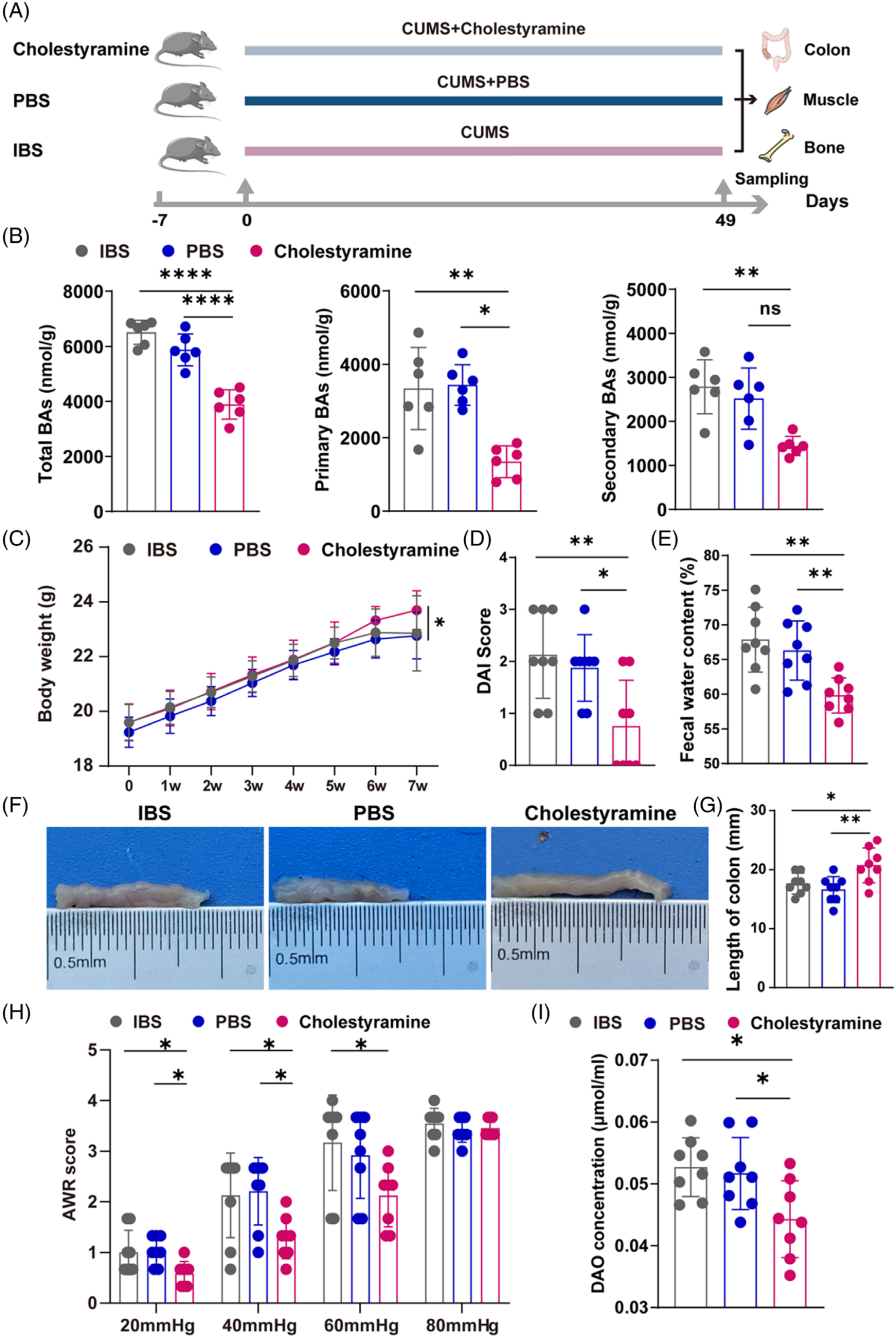
Cholestyramine alleviates IBS phenotypes in IBS mice. (A) Schematic representation illustrating the experimental design. (B) Quantification of total BAs (left), primary BAs (middle) and secondary BAs (right) in the faeces of mice after cholestyramine treatment (*N* = 6). (C) Body weight change during the 7‐week experimental phase (*N* = 8). (D) DAI score evaluation (*N* = 8). (E) Faecal water content evaluation (*N* = 8). (F,G) Representative images (F) and quantitative analyses of colon (G) (*N* = 8). (H) AWR score evaluation in mice (*N* = 8). (I) DAO content detection in mice (*N* = 8). Values are represented as the average ± standard deviation. The significance level (p value) was determined through one‐way ANOVA. *p < 0.05; **p < 0.01; ****p < 0.0001.

The elevated BA levels identified in IBS mice prompted us to explore whether mitigating BA content could alleviate IBS symptoms. To this end, mice underwent administration of either PBS or cholestyramine, a non‐absorbable BA sequestrant known for its BA‐binding capacity, concurrent with IBS induction (Figure 5A). Subsequent faecal BA analyses revealed a substantial decrease in both total and primary BAs following cholestyramine treatment, corroborating the compound's efficacy (Figure 5B, Figure S3). Furthermore, post‐cholestyramine administration, mice exhibited a modest increase in body weight relative to both IBS and PBS groups (Figure 5C). Notably, DAI scores (Figure 5D) and faecal water content (Figure 5E) were markedly reduced in cholestyramine recipients. Histological examination of colon tissue revealed an elongated colon in the cholestyramine group, contrasting with the other two cohorts (Figure 5F,G). Moreover, we found the mitigated IBS in the cholestyramine‐gavage mice with a significant reduction of AWR score (Figure 5H). In assessing the impact of cholestyramine on intestinal integrity, we noted a reduced serum DAO concentration in treated mice compared to IBS and PBS groups, suggesting attenuated intestinal trauma and bolstered gut barrier functionality (Figure 5I). Collectively, these findings underscore the therapeutic potential of BA sequestration in attenuating IBS phenotypes and fostering intestinal health.

### 3.6 Administration of cholestyramine ameliorates bone and muscle loss

**FIGURE 6:**
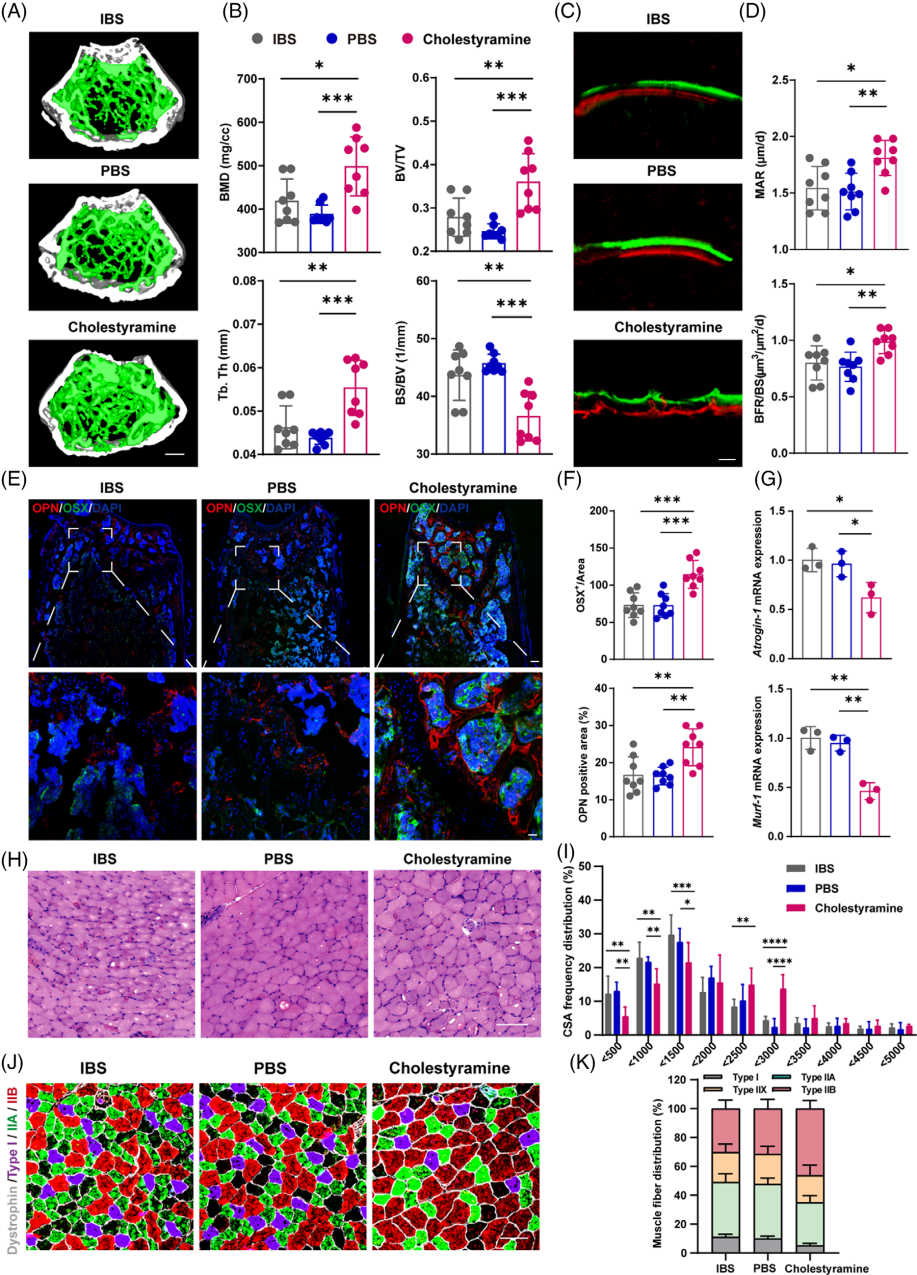
Cholestyramine alleviates IBS‐induced bone and muscle loss in mice. (A) Representative μCT images of distal femoral metaphyseal trabecular bone. (B) Quantitative analysis of bone mass, including BMD, BV/TV, Tb. Th and Tb. N (*N* = 8). Scale bar, 500 μm. (C,D) Representative images (C) and quantification (D) of new bone formation assessed by dynamic histomorphometric analyses (*N* = 8). Scale bar, 25 μm. (E,F) Representative images (E) of OSX (green) and OPN (red) immunostainings and quantification (F) of OPN^+^ and OSX^+^ area on distal femurs (*N* = 8). Scale bar, 100 and 25 μm, respectively. (G) mRNA Expressions of *Atrogin‐1* and *Murf‐1* in gastrocnemius, tested by qPCR (*N* = 3). (H,I) Representative images of H&E staining in gastrocnemius cross‐sections (H) and frequency distribution of CSA (I) (*N* = 8). Scale bar, 100 μm. (J) Representative immunofluorescence images to visualize specific types of muscle fibres. Type I (purple), type IIA (green), type IIX (not shown) and type IIB (red) (*N* = 8). Scale bar, 100 μm. (K) Fibre type composition (*N* = 8). Values are represented as the average ± standard deviation. The significance level (*p* value) was determined through one‐way ANOVA. *p < 0.05; **p < 0.01; ***p < 0.001; ****p < 0.0001.

Given BAs' protective capacity in IBS, we evaluated whether altering BA metabolism could counteract bone and muscle deterioration in IBS‐afflicted mice. Post‐cholestyramine administration, μCT analysis revealed a pronounced enhancement in trabecular bone volume among treated mice (Figure 6A). Consistent with this, we observed higher BMD, BV/TV, Tb. Th and lower BS/BV, indicating the improvement in bone mass (Figure 6B). Subsequent mechanical stress assays further attested to the bolstered bone integrity in the cholestyramine cohort, as evinced by escalated Young's modulus, peak load, rigidity and fracture energy parameters (Figure S4). Regarding bone genesis, dynamic histomorphometric analyses as well as immunofluorescence staining against OSX and OPN showed significant increases in the MAR, BFR (Figure 6C,D) and the numbers of OSX‐ and OPN‐expressing osteoblasts (Figure 6E,F) within cholestyramine‐treated mice.

Muscle assessment through qPCR analysis of gastrocnemius identified subdued *Atrogin‐1* and *Murf‐1* expressions in cholestyramine‐exposed mice, implying muscle atrophy mitigation (Figure 6G). Corroborating the gene expression changes in muscle, H&E‐stained gastrocnemius sections identified enhanced sarcolemmal preservation, amplified CSA and enlarged minimal Feret diameters post‐cholestyramine (Figure 6H, Figure S5). The CSA distribution further shifted rightward for the cholestyramine group relative to controls (Figure 6I), indicative of diminished muscle impairment and fibre enlargement. Additionally, muscle fibre composition appeared reoriented in cholestyramine recipients, with immunofluorescence denoting escalated type IIB fibre prevalence at the expense of type I fibres, suggesting a glycolytic fibre inclination over oxidative counterparts (Figure 6J,K, Figure S6). Taken together, these observations advocate for cholestyramine's therapeutic prowess in rescuing the bone and muscle loss in IBS mice.

### 3.7 Cholestyramine alleviates bone and muscle loss by adjusting gut microbiota composition

**FIGURE 7:**
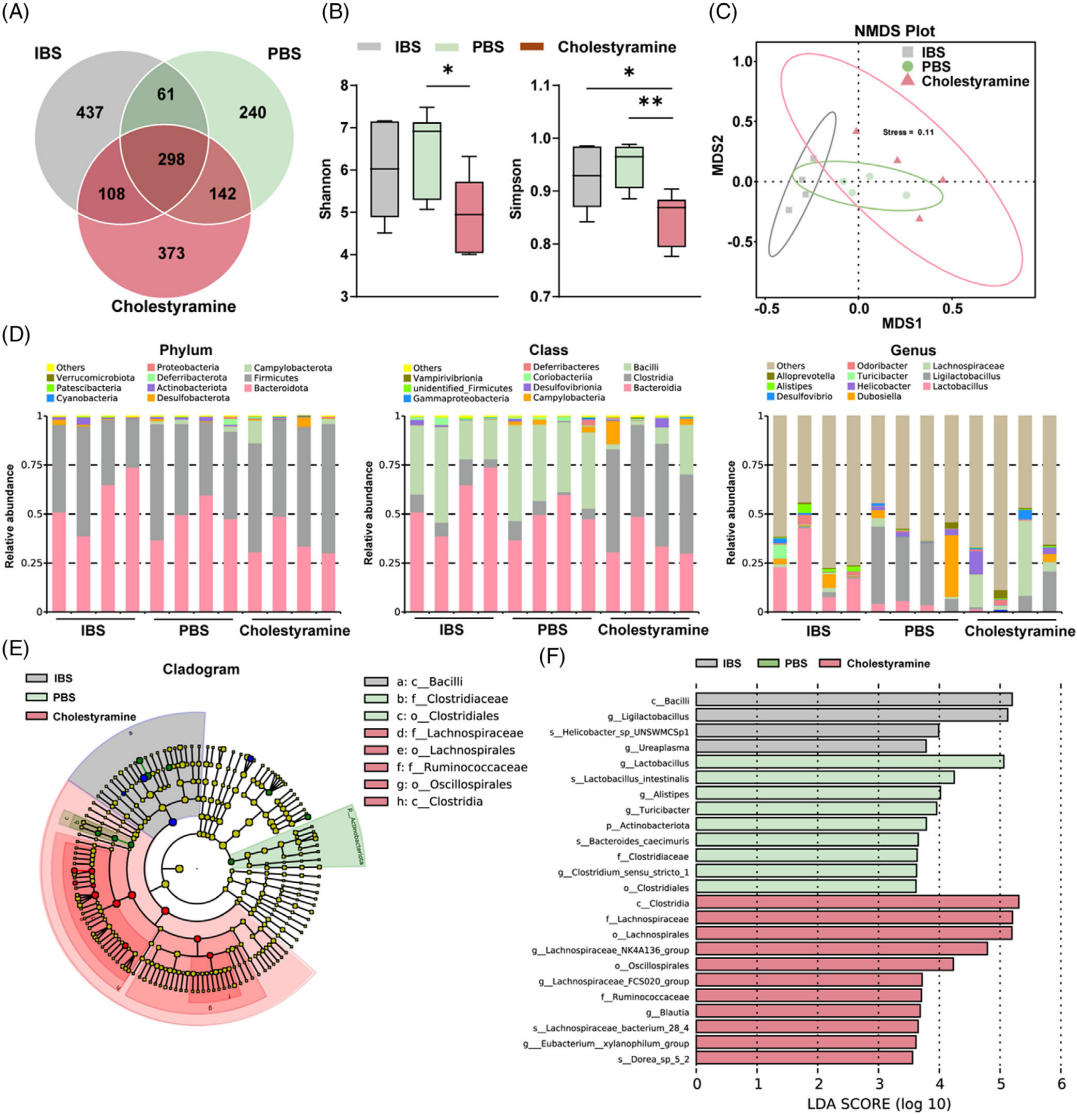
Cholestyramine regulates gut microbiota composition in IBS mice. (A) Venn diagram analysis of gene numbers detected in three groups. (B) The box plot illustrates *α*‐Diversity using the Shannon and Simpson indices. (C) Principal Coordinate Analysis (PCoA) of *β*‐diversity at the phylum tier is conducted via a Bray–Curtis matrix comparison for three groups. (D) Structure plot of the relative faecal bacterial abundances in phylum, class and genus‐level based on Bray–Curtis distance. (E,F) Analysis of Cladogram generated from LEfSe (E) and LDA score (F) across different taxa levels. Values are represented as the average ± standard deviation. The significance level (*p* value) was determined through one‐way ANOVA.

In light of emerging evidence supporting a gut‐musculoskeletal axis, we investigated the effects of cholestyramine on gut physiology and microbiota in IBS‐induced mice, and its subsequent impact on bone and muscle health. Our in‐depth metagenomic analysis of faecal samples from cholestyramine‐treated, PBS‐treated and untreated IBS‐induced mice revealed significant discrepancies in species abundance. This was evidenced by Venn diagram variations (Figure 7A), shifts in *α*‐diversity (Shannon and Simpson index) (Figure 7B) and alterations in *β*‐diversity via PCoA (Figure 7C). Cholestyramine‐treated mice exhibited a partial restoration of microbiota composition at multiple taxonomic levels compared to controls. Phylum‐level analysis showed that cholestyramine significantly increased the abundance of *Firmicutes*, *Campylobacterota* and *Desulfobacterota*, while reducing *Bacteroidota* and *Actinobacteriota* (Figure 7D, left). At class level, we observed increases in *Clostridia*, *Campylobacteria* and *Desulfovibrionia*, while decreases in *Bacilli*, *Bacteroidia* and *Coriobacteriia* (Figure 7D, middle). Genus‐level changes included heightened presence of *Lachnospiraceae*, *Ligilactobacillus*, *Helicobacter* and *Desulfovibrio*, with reduced counts of *Lactobacillus*, *Dubosiella*, *Alistipes*, *Turicibacter* and *Odoribacter* (Figure 7D, right). Subsequent LEfSe analysis and LDA scores indicated that cholestyramine treatment resulted in higher abundances of *Clostridia*, *Lachnospiraceae*, *Ruminococcaceae* and *Eubacterium* (Figure 7E,F). Notably, we observed an increase in microbiota associated with BA metabolism, suggesting a potential positive feedback wherein increased BA biotransformation directly leading to reduced gut BAs.

Collectively, our findings imply that BA sequestrants may shield against intestinal damage and gut microbiota dysregulation prompted by IBS. This highlights a potential interplay among BAs, gut microbiota and intestinal health. Consequently, cholestyramine emerges as a promising therapeutic avenue for mitigating bone and muscle loss in IBS patients, facilitated by its regulatory effects on gut functionality and microbiota composition.

## 4 DISCUSSION

Muscles and bones are not literally connected but are highly intertwined and interdependent. The biology and homeostasis of these two tissues are intricately connected at almost all levels of living such as biomechanical, cellular, paracrine, neuron and so on. Increasing evidence shows that the compromise of one tissue in bone and muscle can trigger a decline in the other, so called osteosarcopenia.^24^ Former studies suggested that osteosarcopenia has a close link with chronic inflammation status such as obesity, type 2 Diabetes mellitus, polycystic ovary syndrome and aging process.^25^, ^26^ Notwithstanding, research has mostly emphasized the singular study of bone or muscle pathology as their investigation in musculoskeletal system. It remains unclear that under what specific circumstances bone and muscle loss may easily happen and how it happened. Our study provides evidence that IBS mice can display osteosarcopenia phenotype and cholestyramine ameliorate IBS‐associated bone and muscle loss via attenuating BAs in gut and modulating gut microbiota composition.

Although some clinical researches indicate that IBS could be a risk factor for osteoporosis, the evidence that sarcopenia is linked with IBS is limited.^27^ Additionally, the composition of gut microbiota in the development of bone and muscle loss in IBS condition is unknown. Our study firstly observed two‐pronged effects of osteoporosis and sarcopenia in IBS‐induced mice and delineated microbiota map in the development of osteosarcopenia. We employed a chronic unpredictable mild stress (CUMS) model to mimic human irritable bowel syndrome. The CUMS model established successfully that the mice are characterized by diarrhoea, weight loss, impaired intestinal barrier function and augmented visceral hypersensitivity. Previous studies have reported diminished bone mass and altered microarchitecture in mice with inflammatory bowel disease (IBD),^28^, ^29^ while there is insufficient information of bone loss in mouse model with IBS. We utilized μCT analysis to demonstrate bone structure and further observed mechanical properties of bones in IBS‐induced mice. We further investigated bone formation ability via immunostaining of osteoblasts. For muscle, we conducted several researches including H&E staining and qPCR of muscle atrophy indicators. Additionally, we demonstrated the transformation of oxidative fibres (type I) to glycolytic fibres (type IIB) in IBS‐induced mice, suggesting an alteration in muscle metabolism.

The gut‐bone axis and gut‐muscle axis theory are well established that intestinal microbiota can influence nutrient metabolism and host immune response, resulting in an impact on bone and muscle at distance.^30^, ^31^ To further illustrate the link between gut function and bone and muscle phenotypes, we conducted metagenomic analysis and found that the gut microbiome was altered after IBS induction. Additionally, it is intriguing that the abundance of bacteria which are competent in BA biotransformation was altered, indicating the potential role of BAs in the development of bone and muscle loss. We confirmed our hypothesis through examining BA levels in the faeces of IBS mouse models and human cases. Notably, an increase of primary BAs, was observed in the IBS group. Previous experiments have validated that the elevation of primary BAs was highly associated with diarrhoea and IBS in human. Oral administration or infusion of CA and CDCA and infusion of primary BAs into colon could accelerate colon motility, inducing diarrhoea.^32^ In addition, it has been demonstrated that there is a positive association between the colonic transit rate of IBS‐D patients and primary BAs in faeces.^33^

It is the metabolites of gut microbiota in the colonic lumen that contribute to the crosstalk between microbe and host physiology.^34^ For example, scientists illustrate that *Firmicutes* stimulate the production of colonic serotonin which is involved in osteoporosis and irritable bowel syndrome.^35^ Gut microbiota is imperative to the BAs metabolism in gastrointestinal tracts.^36^ In the first instance, conjugated BAs secreted into the intestine can be hydrolyzed to free BAs under the action of bile salt hydrolase (BSH) enzymes in various bacteria. And this is a prerequisite for further BAs metabolism by the microbiome. Subsequently, primary BAs, which are synthesized directly in hepatocytes from cholesterol, are converted to the secondary BAs in the lower part of the small intestine and colon by 7α dehydroxylation of some flora.^37^ BSH distributes widely at the phylum level including *Firmicutes*, *Bacteroidetes* and *Actinobacteria* in humans,^38^ while the capability to convert primary BAs to secondary BAs is limited to only a small number of species, mainly in *Clostridiales*, with an overwhelming majority of the strains belonging to *Ruminococcaceae*, and a marginal part belonging to the *Lachnospiraceae* and *Peptostreptococcaceae* families.^39^ In our study, we observed significant decreases in the abundance of *Ruminococcaceae*, *Firmicutes*, *Eubacterium* and *Clostridium* which are associated with BAs 7α/7β‐de‐hydroxylation and deconjugation. Consistent with the microbiota alteration, we found out increases in primary BAs including CA and CDCA and decreases in secondary BAs including DCA, LCA and UDCA in the faecal sample of IBS group, indicating impaired BAs transformation function from primary BAs to secondary BAs.

Previous studies in our team have revealed the same result in IBS patients that BAs were significantly increased.^23^ Therefore, building upon the observation above, we decided to find out whether BAs could be utilized as a potential therapeutic intervention for bone and muscle loss. Our findings demonstrated that BA sequestrants administration ameliorate symptoms of bone loss and muscle loss, while it made the microbiota landscape partially reversed to normal type. It is noteworthy that we observed increases in the abundance of BAs associated microbiota. The results support supposition that there are reciprocal regulations between the microorganism and BAs.^40^ Despite BAs' role in gastrointestinal system, BAs can also act indirectly by binding to the associated receptors and participate in a series of systematic physiological activities. For instance, researchers found that CA and DCA, in a form dependent on TGR5 expression, induce an atrophic condition in skeletal muscle fibres, concomitant with increased levels of oxidative stress and protein catabolic pathways.^41^ Moreover, it was revealed that IBS have a bilateral effect in bone health. Some scientists claimed that BAs enhanced osteoblastic differentiation through binding with Farnesoid X receptor^42^ and serum BA levels have been proved to have a positive correlation with BMD in postmenopausal women.^43^ However, some researchers argued that BAs are risking factors of osteoporosis in primary sclerosing cholangitis (PSC), type 2 diabetes.^44^, ^45^ While our research marks a significant stride in this direction, the intricate mechanisms through which BAs affect bone and muscle health, particularly under IBS‐induced stress, remain elusive. This knowledge gap signifies the need for extensive future studies focusing on the systemic roles of BAs and the underlying molecular crosstalk in the gut‐musculoskeletal axis.

**FIGURE 8:**
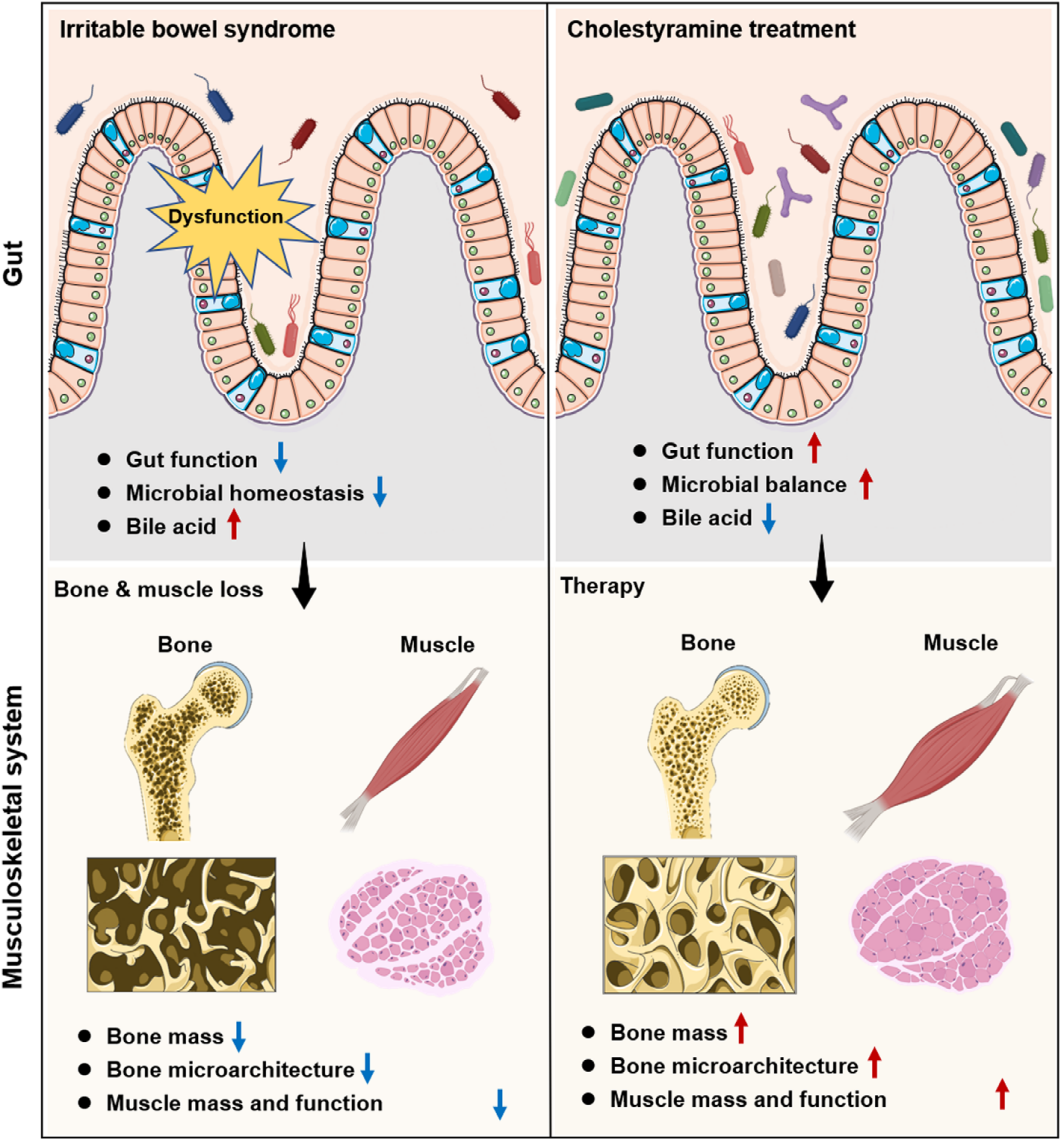
Schematic diagram showing the role of cholestyramine in ameliorating bone and muscle loss in irritable bowel syndrome. Irritable bowel syndrome (IBS) causes gut dysfunction, reflected by a change in bowel habits alongside abdominal discomfort. This leads to osteosarcopenia, characterized by bone loss and muscle deterioration. Bile acids are notably increased, and the taxa for bile acid transformation are decreased during this process. Administration of cholestyramine lowers the bile acids content and restores this gut microbiota balance, which subsequently results in an alleviation in bone and muscle loss under IBS condition.

In conclusion, our study stands as a vanguard exploration illustrating the relevance between IBS, osteosarcopenia and BAs. Given that, there is a paucity of targeting treatment of bone and muscle loss and pharmaceutical interventions addressing sarcopenia are still in development, our study paves the way for future translational research aiming to develop BA sequestrants‐based approaches to treat bone and muscle loss, particularly in patients with IBS. Further investigations exploring the underlying mechanisms and conducting clinical trials are necessary to fully elucidate the therapeutic potential of cholestyramine in bone and muscle loss management (Figure 8).

## AUTHOR CONTRIBUTIONS

MC, WW and PT conceived and designed the study. MC and WW performed most of the experiments and analysed and interpreted all the data. MC, SG, JS, JG and YZ assisted most of animal experiments. XS, DC, FC and JC performed validation experiments under the supervision of KY. JL, QZ, TZ, HZ and XH helped with the sample information analysis. MC, WW and YL wrote the manuscript with inputs from PT and KY. All authors read and approved the final article and take responsibility for its content.

## CONFLICT OF INTEREST STATEMENT

The authors declare that they have no known competing financial interests or personal relationships that could have appeared to influence the work reported in this article.

## Supporting information


**Data S1:** Supporting Information.

## Data Availability

The data that support the findings of this study are available from the corresponding author upon reasonable request.
