# Supplementary material for: Cholestyramine alleviates bone and muscle loss in irritable bowel syndrome via regulating bile acid metabolism
Source: Cell Prolif. 2024 Mar 25;57(8):e13638. doi: 10.1111/cpr.13638 (PMC11294414; doi:10.1111/cpr.13638)
Supplement: Supplementary file 1 — Data S1: Supporting Information. [file CPR-57-e13638-s001.docx]

**Cholestyramine alleviates bone and muscle loss in irritable bowel syndrome via regulating bile acid metabolism**

Ming Chen^1,2,#^, Wei Wei^3,#^, Yi Li^1,2^, Siliang Ge^1,2^, Junmin Shen^1,2^, Jiayu Guo^3^, Yu Zhang^3^, Xiang Huang^1,2^, Xinyu Sun^1,2^, Dongliang Cheng^1,2^, Huayong Zheng^1,2^, Feifan Chang^1,2^, Junyu Chen^1,2^, Jiang Liu^4^, Qinxiang Zhang^1,2^, Tianjunke Zhou^1,2^, Kang Yu^3,*^, Peifu Tang^1,2,*^

^1^ Senior Department of Orthopedics, The Fourth Medical Center of Chinese PLA General Hospital, Beijing, 100853, China.

^2^ National Clinical Research Center for Orthopedics, Sports Medicine & Rehabilitation, Beijing, 100853, China.

^3^ Department of Clinical Nutrition, Peking Union Medical College Hospital, Chinese Academy of Medical Science and Peking Union Medical College, Beijing 100730, China.

^4^ Department of Orthopedic Surgery, Second Affiliated Hospital of Harbin Medical University, Harbin, 150001, China.

Correspondence:

Peifu Tang, PhD

Director, Department of orthopedics, Chinese PLA General Hospital,

Director, National Clinical Research Center for Orthopedics, Sports Medicine & Rehabilitation

No. 28 Fuxing Road, Beijing, 100853, P. R. China

Tel: 86-10-66938101, Fax: 86-10-6821-2342, E-mail: pftang301@126.com

Kang Yu, MD

Director, Department of Clinical Nutrition, Peking Union Medical College Hospital, Chinese Academy of Medical Science and Peking Union Medical College

No.1 ShuaiFu Yuan, Beijing 100730, P. R. China

Tel: 86-10-69154097, E-mail: yuk1997@sina.com

These authors contributed equally: Ming Chen, Wei Wei


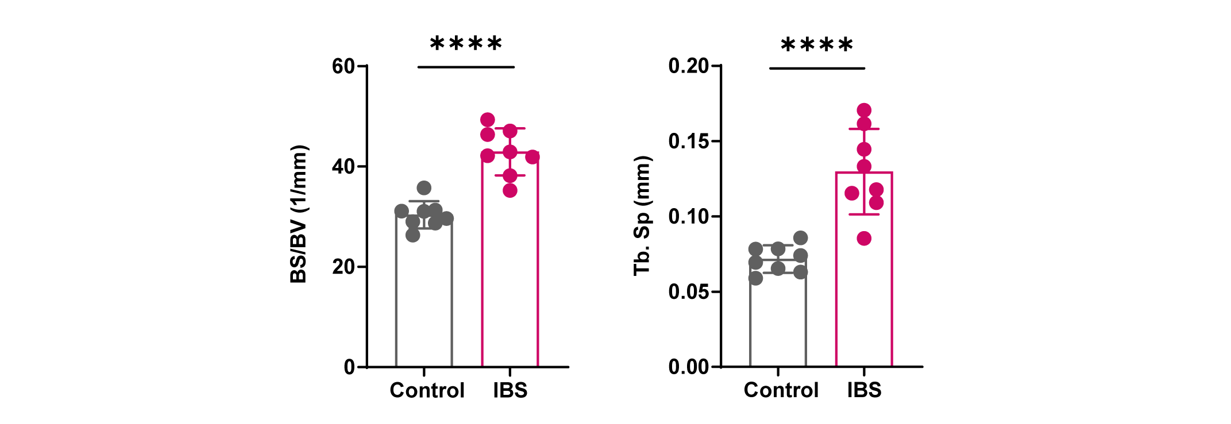


**Supplementary Figure 1. The effects of IBS on bone mass.** Quantification of bone surface area/bone volume (BS/BV) and trabecular spacing (Tb. Sp) (N=8). Values are represented as the average ± standard deviation. The significance level (p value) was determined through a two-sided Welch’s t-test. ****p < 0.0001.


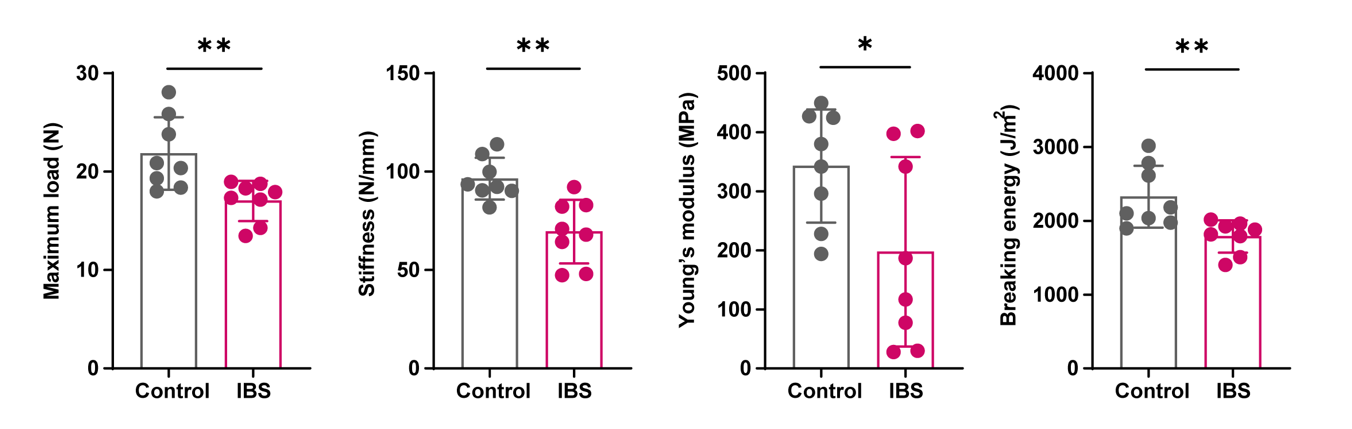


**Supplementary Figure 2. The effects of IBS on mechanical properties of bone.** Quantification of maximum load, bone stiffness, Young’s modulus, and breaking energy (N=8). Values are represented as the average ± standard deviation. The significance level (p value) was determined through a two-sided Welch’s t-test. *p < 0.05; **p < 0.01.


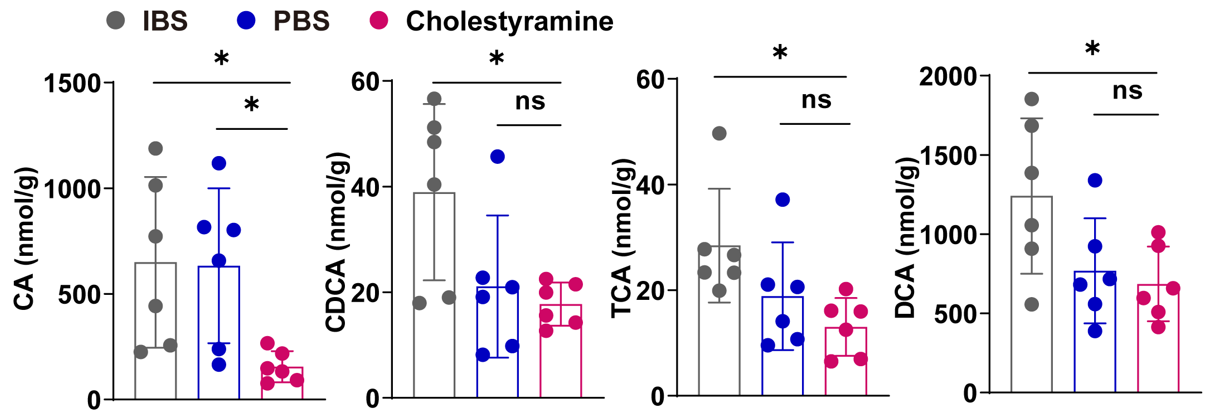


**Supplementary Figure 3. The effects of cholestyramine on bile acid contents.** Quantification of cholic acid (CA), chenodeoxycholic acid (CDCA), taurocholic acid (TCA) and deoxycholic acid (DCA) (N=6). Values are represented as the average ± standard deviation. The significance level (p value) was determined through one-way ANOVA. *p < 0.05.


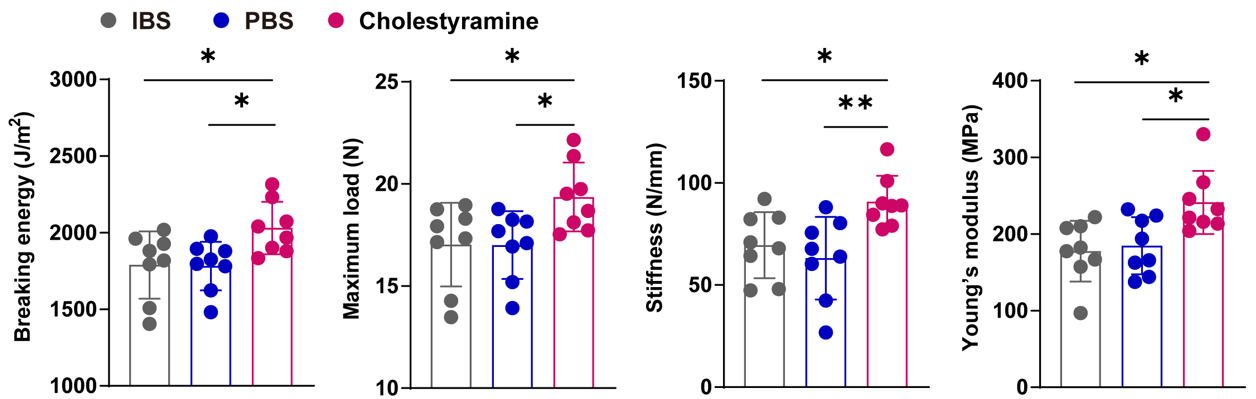


**Supplementary Figure 4. The effects of cholestyramine on mechanical properties. of bone.** Biomechanical analysis of femur, including the breaking energy, maximum load, stiffness, and Young’s modulus (N=8). Values are represented as the average ± standard deviation. The significance level (p value) was determined through one-way ANOVA. *p < 0.05; **p < 0.01.


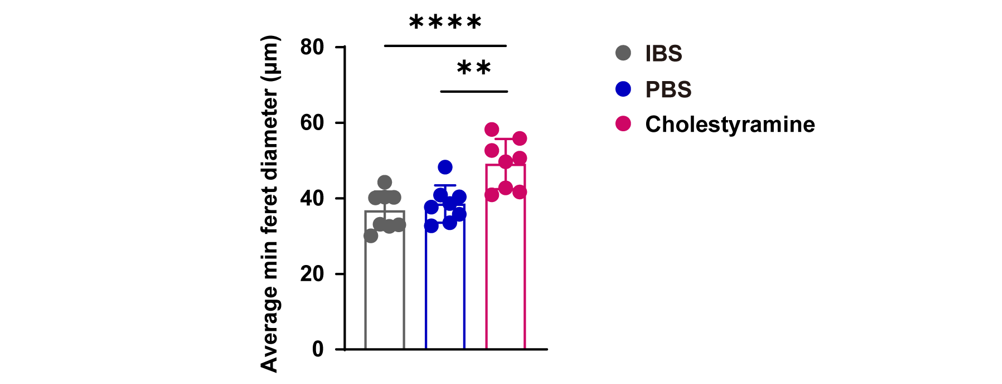


**Supplementary Figure 5. The effects of cholestyramine on muscle fiber diameter in IBS mice.** Quantification of average min feret’s diameter (N=8). Values are represented as the average ± standard deviation. The significance level (p value) was determined through one-way ANOVA. **p < 0.01; ****p < 0.0001.


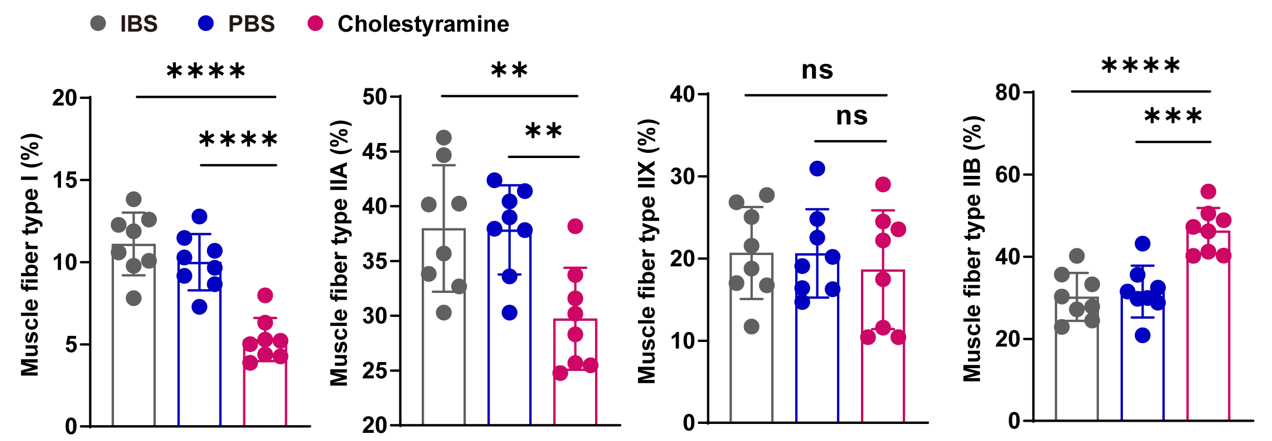


**Supplementary Figure 6. The effects of cholestyramine on muscle fiber type composition.** Quantification of muscle fiber type (type I, IIA, IIX and IIB) (N=8). Values are represented as the average ± standard deviation. The significance level (p value) was determined through one-way ANOVA. **p < 0.01; ***p < 0.001; ****p < 0.0001.

**Supplementary Table 1. Chronic unpredictable mild stress (CUMS)-induced IBS model scheme.**

| **CUMS Protocol** | | |
| --- | --- | --- |
| **Number** | **Projects** | **Specific Operations** |
| 1 | Fasting | Fasting for 12 hours |
| 2 | Water Deprivation | Keep water off for 12 hours |
| 3 | Tilting | Tilt the cage 45° overnight |
| 4 | Wet Pressure | Live in a damp cage for 12 hours |
| 5 | Flash Illumination | Flash shine overnight |
| 6 | Tail-pinch | Clip the tail with forceps or pliers for 5 min |
| 7 | Swimming in cold water | Swim in cold water at 4℃ for 5 min |
| 8 | Bondage | Bondage for 6 hours |
| 9 | Shaking | Shake cages for 15 min |

**Supplementary Table 2. Disease activity index scoring**

| **Score** | **Body weight loss (%)** | **Stool consistency** | **Stool occult blood ^a^** |
| --- | --- | --- | --- |
| 0 | None | Normal | Negative |
| 1 | 1-5 | Soft stools | Positive (+) |
| 2 | 6-10 | Very soft stools | Positive (++) |
| 3 | 11-18 | Watery stools (Diarrhea) | Positive (+++) |
| 4 | ＞18 |  | Visible rectal bleeding |

^a^ Positive (+) indicates color change from light green to green within 10s, while Positive (++) shows color change from green to blue within 30s and Positive (+++) to dark blue immediately according to the instructions of manufacturer (Brybio, Beijing, China).

**Supplementary Table 3. RT-qPCR primer list**

| **qPCR Primer list** | **Forward Primer** | **Reverse Primer** |
| --- | --- | --- |
| *Atrogin-1* | CTTCTCGACTGCCATCCTGGAT | TCTTTTGGGCGATGCCACTCAG |
| *Murf-1* | TACCAAGCCTGTGGTCATCCTG | ACGGAAACGACCTCCAGACATG |
| *β-actin* | CATTGCTGACAGGATGCAGAAGG | TGCTGGAAGGTGGACAGTGAGG |
